# Why Is Rehabilitation Assistance Policy for Children With Disabilities Deviated in Supply-Demand? A Case Study in Mainland China

**DOI:** 10.3389/fpubh.2021.666333

**Published:** 2021-04-07

**Authors:** Cai Yun Qi, Yuan Wang

**Affiliations:** Department of Labor and Social Security, School of Philosophy and Sociology, Jilin University, Changchun, China

**Keywords:** rehabilitation assistance system for disabled children, supply-demand deviation, policy context, exclusion error, inclusion error

## Abstract

Children with disabilities have most potential for salvage rehabilitation, and their rehabilitation results are concerned with their entire life process. Although, the Chinese state has established a targeted Rehabilitation Assistance System for Disabled Children and has expanded the provision of rehabilitation services, a severe deviation between supply and demand remains. Existing studies have focused relatively more on policy content and less on the policy context, at the macro-structural level. However, using the case of the ZW Rehabilitation Center in City J, this study divided the deviation into exclusion errors and inclusion errors, and used the policy context approach to explore the reasons for the deviation. We found that the behaviors of the participants in rehabilitation services exist in a dynamic interaction between the regulatory context, the normative context, and the cognitive context. The joint forces of the three contexts produce both exclusion errors and inclusion errors, which are the underlying reasons for the inaccurate execution of the targeted policy. The results of this research can provide enlightenment for improving rehabilitation policy.

## Introduction

Currently, there are 85.02 million persons with disabilities in Mainland China – the maximum number per nation in the world. Rehabilitation has become one of the most important ways to restore or compensate for disabled people's body functions and improve their quality of life, and China is paying increased attention to such rehabilitation efforts. Since 1988, the state has integrated its rehabilitation program for persons with disabilities into national economic and social development plans to protect those individuals (1). Recently, a brief government document also noted that a new targeted rehabilitation program had been implemented (2). Indeed, “targeted” is a hot word in present-day China, having been derived from the term “targeted poverty alleviation,” which authorities first proposed in 2013. Targeted rehabilitation refers to the provision of rehabilitation services on the basis of the actual needs and difficulties of each disabled individual, through accurate diagnosis and scientific evaluation (3).

In the full plan for rehabilitation of persons with disabilities, rehabilitation for children is the starting point, and is concerned with the children's entire life process. Studies have shown that the most successful time for the rehabilitation is between 0 and 6 years of age, because during that period the brain has the best flexibility, with the best compensatory effects, for restoring an injured brain's structure and function (4). For children, grasping the opportunity of early salvage rehabilitation can effectively avoid or decrease later complications and sequelae, thus, creating opportunities for their education, employment, and social integration in the future. Currently, there are 1.678 million children with disabilities in this age range in China (5). Most of those children are faced with different kinds of risks, including poverty, malnutrition, poor health, lack of family protection, and so on (6), and many have extensive demands for medical rehabilitation and care. To satisfy those demands, *the CPC Central Committee* and the *State Council on Promoting the Development of the Cause of Disabled People (2008)* stated the opinion that salvage treatment and rehabilitation for children with disabilities should be given priority and that salvage rehabilitation projects for poor disabled children by the China Disabled Persons' Federation (CDPF) were to be carried out. Beginning in 2018, guided by the concept of targeted rehabilitation and overseen by *the State Council on the Establishment of a Rehabilitation Assistance System for Disabled Children*, rehabilitation services have been provided for children aged 0–6^1^ years who have a disability of vision, hearing, speech, limb, intelligence, or who have autism. The content of the services includes operations, assistive devices configuration, and rehabilitation training, with the goals of reducing dysfunction, improving functional status, and raising the individual's ability for self-care and social participation (7).

The interactions between service providers and service users, reported from their experiences of providing and obtaining welfare, can establish a relational understanding of policy (8). From the position of service providers, the Rehabilitation Assistance System for Disabled Children (RASDC) is an inclusive policy that is based on civil rights, and it can effectively address the rehabilitation barriers. However, from the position of service users, this service has not had a strongly positive effect. The rehabilitation provision rate is low, remaining far from the goal of targeted rehabilitation. Survey data have identified that after the implementation of the policy, the supply rate of the service is only 20.33%, whereas, the demand rate is approximately 27% (9). After China has provided a generous rehabilitation service for disabled children, why is there still a severe supply-demand deviation?

To answer that question, this study distinguished between two kinds of deviations and investigated them using the perspective of the policy context. Specifically, studies evaluating the effectiveness of social welfare programs have found that two deviations commonly occur in attempts to “target” public expenditures for poor people in poverty reduction policies. One is an “exclusion error” (also called “undercoverage,” or “failure to reach the prime objective”), in which some subjects are eligible to receive benefits but do not receive them, and the other is an “inclusion error” (also called “leakage,” or “excessive coverage”), in which those who receive benefits are not eligible to receive them (10, 11). In trying to classify the supply-demand deviation in rehabilitations services, attention has tended to focus on one error and to overlook the other, and that single-error focus has led to a narrow understanding of the supply-demand deviation. In our research, we too found the existence of these two errors in the implementation process of the targeted RASDC. However, relevant extant research on this area is scarce. To explore the reasons for exclusion errors and inclusion errors, we used the policy context, and specifically the regulatory, normative, and cognitive contexts, as a new perspective. That approach gives a much more comprehensive framework that can capture various aspects of the ideology, political interests, cultural norms, social knowledge, rules and regulations, and other features that could have a considerable impact on the administrative process (12, 13). This study, conducted in the local environment of present-day Mainland China, sought to reveal the supply-demand deviation of the RASDC in China, and to enrich the academic community's understanding of how the local political and cultural contexts change the established goals of policy.

## Literature Review

In China, the research on rehabilitation has been conducted mainly in the field of medicine, and studies on the rehabilitation policies for disabled children have been carried out only recently. The proposal in China of the RASDC in 2018 was an important watershed event, and the topic gradually became a hot button in academic circles. Although, some scholars have conducted ongoing research on the issues of rehabilitation services for disabled children, most of them have focused on only the service content. From that approach, the findings have shown that the inaccurate implementation of the RASDC has been caused by ideas, methods, and specific contents of provision.

The first aspect of implementation is in regard to the idea of provision, of putting the child first, and maximizing the benefits for the child, but at present in China that idea is not met with consensus among all, and the principle that the state is the supreme guardian and protector of the child is not established. As a result, the RASDC efforts have mainly been survivor-oriented, and have been lacking in inclusive and development-oriented policies (14). Studies have shown that, although state fiscal expenditures are increasing, a lack of funds, the use of strict criteria for rehabilitation assistance, and the problem of rehabilitation demands not being included in the RASDC are still major dilemmas (15).

Second, regarding the methods for provision, medical rehabilitation services for children with disabilities are currently provided by designated agencies, mainly in the form of “government procurement services.” Among those services, imperfect service skills in agencies are the most important factor, and that problem produces an exclusion error. Poor rehabilitation service networks, lack of professional staff, low professional levels, and slow updating of rehabilitation technologies have all affected the effectiveness of provision of rehabilitation services (16–18).

Finally, some research has suggested that the RASDC policy lacks supporting policies (19). The RASDC is mainly aimed at helping poor households to solve the problem of excessive costs in medical care and rehabilitation for children with disabilities. However, those households are faced with more severe parental pressures in the process of rehabilitation, compared with households with disabled people of other ages (20, 21). The long-term care needs of disabled children gradually increase the physical and emotional burdens within families, and those burdens cannot be solved by insufficient support services. That increasing pressure, in turn, can lead the recipients to voluntarily abandon rehabilitation services (22). Additionally, those service providers make medical rehabilitation the main focus, and they ignore the universal demands of community-based and family-based rehabilitation (23, 24).

Those reviews have demonstrated that many exclusion errors occur from the ideas, methods, and specific contents of rehabilitation provision. As a result, a large number of rehabilitation demands of children with disabilities are not satisfied effectively, and that outcome is far from the goal of targeted rehabilitation. The causes of exclusion errors include such main characteristics as insufficient government financial support, a low supply of rehabilitation services, poor quality of rehabilitation services, uneven distribution of services, lack of equipment, large gaps in rehabilitation professionals, inadequate support systems, and so on (19, 25–27). To solve those barriers, government responsibility should be clarified – that is, the stated goal should include such items as the provision of additional services by increasing government fiscal spending, mobilizing social forces, and expanding the participation space of volunteers (28, 29). It is also important to improve rehabilitation technology and increase the delivery of support services, such as, medical screening, exercise rehabilitation, medical and educational integration, community-based rehabilitation, and care support, among others (30–33).

The discoveries made through the service content approach have attracted widespread attention from scholars, but that is a small portion of the policy context, and it can only find exclusion errors. In addition, the existing literature overemphasizes exclusion errors caused by the policy makers and direct providers. In reality, however, policy implementation is always the interaction between individual factors and environmental factors, and that interaction is exactly what the policy context approach emphasizes. From the policy context approach, we found two types of errors – exclusion errors (with additional, different expressions compared with those in the existing research) and inclusion errors – both of which have led to inaccurate results from the RASDC.

Furthermore, the two universally adopted research methods are limited. Existing studies have mainly applied quantitative methods to measure rehabilitation needs (9, 26, 34), along with a few in-depth interviews (18). In regard to the quantitative methods, field research has found that a large number of children with disabilities and their parents are in a passive state of acceptance, and they are not clearly informed about rehabilitation policies and their own needs, so that accurate statistics cannot be obtained by questionnaire. In regard to the in-depth interviews, the studies have focused only on households that have children with disabilities and have not really outlined the entire process of implementation and identified areas where the error occurred. In response, this study, by using a case study method, analyzed behavioral characteristics, and welfare choices of welfare providers and users in specific fields, to answer the question of how the policy context causes well-targeted rehabilitation programs to be implemented inaccurately.

## Theoretical Framework and Method

### Theoretical Framework

Social policy's “goal-result” is not a simple linear relationship, and complex mechanisms of intermediate phases – policy implementation systems – cannot be ignored. Some seemingly unrelated events in implementation can interfere with the expression of policy objectives, resulting in an imperfect correspondence between the policies and the services actually provided. What factors can affect policy implementation? From Pressman and Wildavsky's research in 1973 (35), various scholars have investigated the question of influential factors on policy in different ways, including factors in society, economy, technology, and the political environment, among others (36). Hill and Hope offered a detailed explanation, pointing out that the dependent variables that affect the outcome of implementation include policy characteristics, policy formation, vertical public administration, influences on implementation agency responses, horizontal inter-organizational relationships, the impact of responses from those affected by the policy, and the environment or policy context (37). In studying policy implementation in the third world, these factors are resummarized as two elements: the content of the plan and a given political-social context (13). Various scholars have all revealed and affirmed that contexts such as, the legal system, culture, social norms, and the values of an organization have a far-reaching influence on policy implementation. However, in studies on the execution of the RASDC, environmental factors have been ignored, either intentionally, or unintentionally.

Researchers have employed different ways to conceptualize and operationalize the context in which individuals and organizations exist. Parsons was concerned with social norms (38), whereas March and Simon examined cognitive and normative structures (39). Furthermore, Scott's classic study suggested that regulative, normative, and cultural-cognitive are the key features of a national context (40). That theoretical framework has groundbreakingly integrated three basic elements as the main composition of the institution. After Scott, scholars have given more attention to the micro applications of this macro theory. Among those scholars, Kostova effectively overcame the limitations of past work and constructed a comprehensive framework of the external environment with which to study the specific phenomenon of quality management of public services between different countries (12). In her paper, “regulatory component” refers to the mandatory laws and regulations in a particular national environment that promote or restrict certain types of individual and organizational behaviors. “Normative component” consists of social norms, social responsibility, values, beliefs, and assumptions about human nature and human behavior that are socially shared and are carried by individuals and organizations. “Cognitive component” comprises social knowledge and cognitive categories that are widely shared in a country, including the cognitive programs that people use when choosing and interpreting information (12). Although, Kostova studied the particular issue of quality management, the approach that she applied is generalizable and could be used in various studies concerned with other issues.

Well-targeted rehabilitation programs for disabled children will not achieve their goals because of a specific policy context. This study, drawing on the theoretical framework of policy context and taking Chinese local knowledge into full account, anchors these three factors – the regulatory component, the normative component, and the cognitive component – into an investigation of the provision issues of the RASDC. We focused on the complex interactions of local governments, service agencies, and children with disabilities, through their maintenance of the political power, emphasis on self-interest, and reinforcement of the negative role of the disabled, and we constructed a supplementary and comprehensive perspective for understanding policy implementation. We argue that the policy context approach extends beyond the dualism of the supply-oriented model and the demand-oriented model, and we seek to provide adequate evidence regarding how these interactions produce both an exclusion error and an inclusion error in connection with the targeted RASDC. Although, each subcontext involves the participation of multiple subjects, in China's political-social context, the RASDC has been implemented *via* the process of the state distributing tasks through top-down orders and the rehabilitation agencies providing the services. In that light, we define the regulatory context as mainly the laws and regulations of organization, distribution, constraints, and incentives by the local governments that are responsible for implementing the RASDC. The normative context is embodied in the values of social norms and social responsibility that rehabilitation agencies follow when allocating and delivering specific resources. The cognitive context refers to the long-term-disabled culture in China, and the fact that individual cognition and social cognition of the RASDC by families with disabilities and the public have a vital impact on behavioral choices.

**Figure 1:**
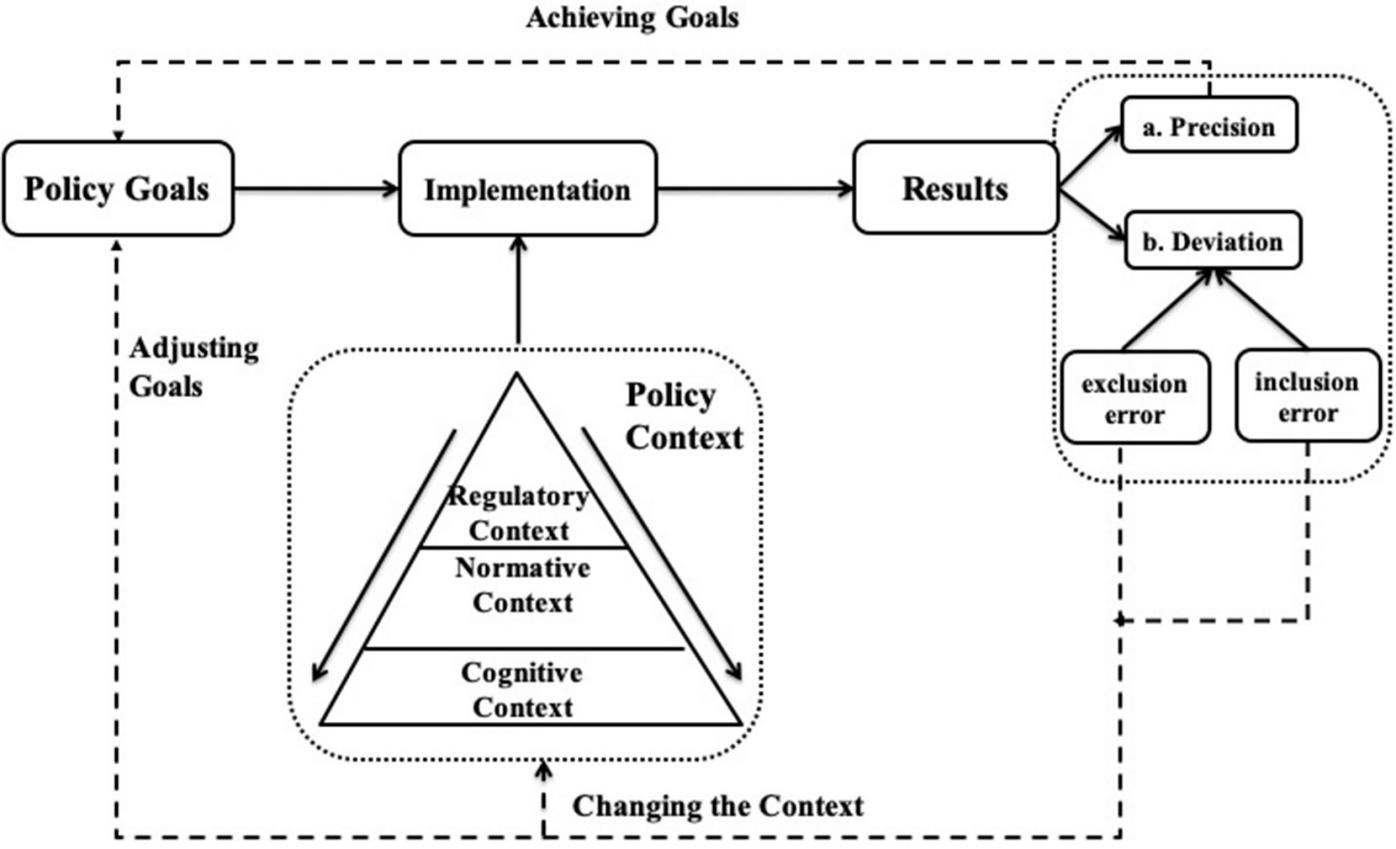
The policy context and its deviations in RASDC implementation.

The research concept for this study is shown in Figure 1. In the implementation stage of the targeted RASDC, the three subelements of regulatory context, normative context, and cognitive context coexist and have a vital impact on the behavior of related individuals and organizations. These lead to two kinds of implementation results: (a) precise implementation – in other words, achieving the policy goals, and (b) deviant implementation, or failure to achieve the policy goals. There are two forms of deviation, called exclusion errors and inclusion errors. To solve them, it is necessary not only to adjust the inappropriate policy objectives, but also through various ways to change any existing unfavorable environment of policy implementation. This two-pronged approach is the key to the problem-solving.

### Methods

We used a qualitative study method and selected ZW in City J as the main research site. We collected ethnographic data by participatory observation, in-depth interviews, and a review of government documents, statistical yearbooks, and other files.

#### Field Access and Sampling

City J, the provincial capital of Province J, is situated in a rich coastal area of eastern Mainland China. Due to political factors, City J is often the first pilot area for the policy practice by the CDPF. In 2013, the city launched a salvage rehabilitation program for children with disabilities. This city, over time, has moved into the fore front of all of China's cities, and it has rich practical experience.

The field work portion of the study started in the summer of 2020. While visiting the rehabilitation agency, the authors gained access to a list of the names and basic information for 51 designated agencies in City J for the RASDC that had been newly established in 2018. Among the 51 agencies, 49 were private non-enterprise units (non-profit organizations). The ZW Child Rehabilitation Center and ZW Chinese Medicine Clinic (really one agency, with different services; hereinafter referred to as “ZW”) was the one with the largest number of disabled children in its services, the largest number of different kinds of disabled children (70 with autism, 90 with mental disability, and 20 with cerebral palsy), and the maximum age range of disabled children (aged 0–17 years). It was also the first designated rehabilitation assistance service agency, approved in 2013, and it has a rich level of experience. Therefore, we selected ZW as the primary research site.

The founder of the ZW organization, after a business failure in 2003 and with no access to converting to Buddhism, decided to make use of his own traditional Chinese medicine skills to do something good for the country and the people, so he established ZW in 2006. The ZW organization developed rapidly by making full use of the characteristic elements of Chinese medicine: acupuncture, massage, medicinal diet, and other traditional Chinese medicine therapies. At present, ZW is a rehabilitation center for cerebral palsy, mental disability, autism, hyperactivity disorder, hemiplegia, and paraplegia, for which it integrates rehabilitation, education, and family-based rehabilitation guidance. It is now engaged primarily in exercise training, homework training, speech training, sensory training, physical therapy, music therapy, guided education, special education, social work, medicinal meals, medicine baths, a spa, massage, acupuncture, near-infrared brain function imaging technology, and other treatment items. The agency is situated in a relatively marginal area of an urban zone in District B, City J, with convenient traffic and low rent, and it covers an area of 2600 square meters. Currently, it has two Chinese medical rehabilitation specialists, 30 therapists, and 22 teachers for special education.

At present, ZW can hold 180 children with disabilities. In July and August, 2020, when we did the research, there were only 139 children at ZW (because of COVID-19, some families had limited movement). The organization primarily receives children with disabilities who live in District B, but some children from other provinces, cities, and districts are also attracted by its reputation. The children range in age from 0 to 17 years, and they have different types and degrees of disability. Their family backgrounds also differ from each other, but they have one thing in common — all are children with disabilities.

#### Data Collection and Data Analysis

With the help of the head of ZW, we collected ethnographic data in July and August 2020. We used three different methods: participatory observation, in-depth interviews, and a review of government documents, statistical yearbooks, and other files.

To begin, the first author had a nearly one-month-long intense participatory observation period within the ZW organization, closely observing each step, including project-setting, implementation, assessment and adjustment, and so on, in the RASDC. Participating as a university volunteer, the author was responsible for the entire work of the RASDC, chiefly handling the issues for parents of disabled children eligible for assistance and also getting an opportunity to dock with the local Disabled Persons' Federation. During that time, three staff members in the administrative office shared with the author their working experiences, feelings, and attitudes toward the implementation of the RASDC. Data from those interviews also were entered into field records of participatory observation, as an important way of data collection.

Next, we conducted 15 in-depth interviews, each ranging from 30 min to an hour. Our interviewees were divided into two categories: three members in the management team, including the founder and head of ZW, one therapist and the director of the administrative office, and 12 primary caregivers of families with children with disabilities. The caregivers were selected after consideration of factors such as, gender, age, household registration (Hukou), children's age, children's disability type, and willingness to be interviewed. Owing to City J's extended age for rehabilitation services to 17 years, our research was not limited to children 0–6 years old but also included interviewees whose children with disabilities were aged 7–17. Before the interviews, we provided the interviewees with general information related to our research, including the purpose, subjects, process, duration of the study, and the research schedule. The entire research process followed the procedures of the institutional review committee and took into account serious requirements for confidentiality, non-harm, and informed consent of participants. More information about the interviewees can be found in Tables 1–3. The authors completed all the one-on-one interviews in Mandarin or dialects, converted all of the interview recordings into Chinese, word by word, and selectively translated some information into English as required in order to present the findings. For confidentiality, all identifying information was anonymized during translation and transcription.

**Table 1:** Description of research participants – caregivers.

**Case**	**Role of interviewee**	**Age**	**Child's age**	**Child's gender**	**Child's disability type**	**Hukou**
						**(District B)**
Case 1	Mother	30	5	F	Mental disability	Non-local
Case 2	Mother	47	11	F	Mental disability	Non-local
Case 3	Father	38	12	M	Autism	Local
Case 4	Uncle	36	15	M	Mental disability	Local
Case 5	Grandfather	65	6	M	Autism	Non-local
Case 6	Father	32	6	F	Mental disability	Non-local
Case 7	Mother	36	4	M	Autism	Local
Case 8	Mother	31	6	F	Cerebral palsy	Non-local
Case 9	Mother	50	14	F	Mental disability	Local
Case 10	Father	37	7	M	Autism	Non-local
Case 11	Mother	49	10	M	Mental disability	Local
Case 12	Mother	29	4	M	Autism	Local

**Table 2:** General characteristics of the research participants (*N* = 12).

**Variables**		***N* (%)**
Children's age	0–6	58.3%
	7–17	41.7%
Children's gender	Male	58.3%
	Female	41.7%
Children's disability type	Mental disability	50%
	Autism	41.7%
	Cerebral palsy	8.3%
Hukou	Local	50%
	Non-local	50%

**Table 3:** Description of research participants – managers.

**Case**	**Age**	**Gender**	**Educational attainment**	**Job title**	**Years working in ZW**
Case 13	50	M	University	Founder & head	16
Case 14	38	M	University	Therapist	14
Case 15	35	F	University	Director	10

Finally, we also studied government departments guidelines, statistical yearbooks, working documents, and other files related to the RASDC, to supplement our field data. In this investigation, we selected information from multiple sources to improve validity.

We coded the respondents' narratives in two stages: one of open coding and one of theoretical coding. Guided by qualitative analysis techniques (41), we integrated raw data from different sources to generate consistent themes, to reveal the process of implementing the RASDC, and to identify the multiple deviations and their contextual causes in this process. Our purpose was to formulate an extended case study with theoretical and practical meaning (42), that could extend the understanding from the existing literature on the supply-demand dilemma for rehabilitation services and could provide some suggestions for policy practices later. The central administrative work system in China has a high degree of the characteristic of “Isomorphic Responsibility,” so the cases selected in this research had a certain expansibility. We try our best efforts to reduce the impact of the limitations of the case method on the results to improve reliability and validity (43).

## Findings

The RASDC takes place in a specific field, embedded in local institutional situations and constrained by habitus. The results of our empirical survey indicate that in the process of delivering targeted services for children with disabilities, there is wide interaction among local governments, rehabilitation agencies, and families. That interaction exists in a perpetual, dynamic tension between the regulatory context, normative context, and cognitive context. In the following paragraphs, we will discuss (1) the constrained and incentive rules produced by Chinese local governments in the regulatory context, (2) the strong norms and weak social responsibility of rehabilitation agencies in the normative context, and (3) the identity culture of children with disabilities and their families in the cognitive context. Additionally, we will present our analysis of how the three contexts generated errors of exclusion and inclusion, which then led to an inability to execute the RASDC accurately.

### Regulatory Context: Special Executive Rules of the Chinese Local Government

In the targeted RASDC system in present-day China, the local government plays a crucial role in providing services, supervising the operations of agencies, and guiding policy management with official discourses. The RASDC adopts many kinds of local executive rules, from which the target management responsibility system, the administrative subcontract system, and promotion tournaments are the keys to executing policy and are also the root cause of deviant behaviors in the regulatory context.

#### Exclusion of Types in the Target Management Responsibility System

China assigns and executes tasks through its target management responsibility system, which is a system form created in a highly centralized environment. Its concrete manifestation is to decompose and refine the general administrative goals step by step, forming a set of tasks systems as the basis for management, rewards, and punishments of organizations at all levels (44). Indeed, our study showed that the rehabilitation experiences of children with disabilities were extremely closely related to their local Disabled People's Federation, which horizontally is an executive institution that is responsible for the formulation and implementation of all matters concerning persons with disabilities, and which vertically is composed, from the top down, by the China Disabled Person's Federation (CDPF), the Provincial Disabled Person's Federation, the Municipal Disabled Person's Federation, and the District Disabled Person's Federation. Under the target management responsibility system, after the Disabled People's Federation has obtained the tasks of the RASDC from the higher level, then the tasks are refined and assigned to the lower level and finally to rehabilitation agencies, according to the top-down structure. For specific tasks, see Table 4. We found that the Disabled People's Federation in City J divides rehabilitation services into four task indexes, thereby assigning the services to children with autism, with limb disability (cerebral palsy), with intelligence disability, and with hearing and speech disability. For visual disability, only low-vision surgery can be provided, and long-term rehabilitation services for children who cannot recover their vision are lacking. That limitation of services deprives children with visual disabilities of access to targeted rehabilitation services, thus, leading to an exclusion error.

**Table 4:** Allocation of rehabilitation assistance tasks for children with disabilities in City J (2018).

**District**	**Tasks**							**Other tasks**	**Total**
	**Ages 0–9**				**Ages 10–17**	**Corrective surgery**	**Low-** **vision surgery**		
	**Hearing & speech disability**	**Mental disability**	**Cerebral palsy**	**Autism**					
A	8	29	26	26	0	0	0	30	119
B	17	70	18	43	24	0	0	20	192
C	15	50	20	18	0	0	0	10	113
D	14	30	12	13	12	0	0	15	96
E	20	53	24	24	20	4	1	10	156
F	15	20	15	15	10	3	0	5	83
G	17	44	40	8	21	0	0	8	138
H	13	15	16	11	5	6	0	0	66
I	19	30	20	4	5	1	0	0	79
J	15	30	8	8	46	0	0	0	107
K	7	19	13	6	4	0	0	10	59
L	8	45	46	1	12	1	0	0	113
Total	168	435	258	177	159	15	1	108	1,321

In addition, the capacity and preferences of the rehabilitation agencies produce a large number of exclusion errors. The tasks assigned by the target management responsibility system are based not on the number of children with disabilities from District A to L, but on the capacity of rehabilitation agencies to receive children. That is, the capacity is calculated by judging the gross building area, number of therapists, curriculum type, and other factors. The numbers for total tasks in Table 4 are the sums of the capacity of each agency in City J. However, having arisen only recently, private non-profit rehabilitation agencies in China do not have the capability to cover all of the children with disabilities. In 2018, 1,321 children received rehabilitation services, whereas the number of children with disabilities in City J exceeded 5,000. Even more serious is the fact that, according to the materials of 51 rehabilitation agencies, with the exception of general child rehabilitation hospitals, most of the private non-profit rehabilitation agencies focus on children with autism and mental disability, while ignoring other types of disabilities.

#### Exclusion of Regions in the Administrative Subcontract System

Another important rule in China's regulatory context is that of the administrative subcontract system. It is a product of the soul of the “contract system” placed under the hierarchical shell, and in it, tasks assigned by the superiors are to be accomplished through administrative power distribution and economic incentives (45, 46). In the process of contracting out the RASDC's tasks, geographic regions serve as the boundaries. Decisions about which region children with disabilities and their families should obtain the source of their services are made through the unique household registration system (hukou system), in which one can only receive the services with the local hukou, and the location of the hukou is based on the person's personal identity card. In China, the flowing population is 245 million people, within which the cross-provincial flowing population is 85.88 million (47). Despite a lack of data on the migrant population of children with disabilities, it is possible to identify a significant number of such groups. The combination of the administrative subcontract and the hukou system results in an exclusion error based on geographic position in the application of the RASDC, when individuals and families whose place of residence and location of hukou are inconsistent. The participant of case 11 left a deep impression on the interviewer because she cried in the interview. She and her family had lived in Tianjin Province for several decades. The family's rehabilitation service was self-financed during the first 3 years, because their hukou was in City J. In the 4th year, after they learned about the free rehabilitation assistance in City J, the mother rented a room near ZW with her disabled child, which meant that she and the child lived a long distance from her husband and eldest son.

Unfortunately, the boundaries of different services vary. Rehabilitation services for children with disabilities use the province as the boundary, whereas, education services use the city or even the district as the boundary. Children with disabilities who receive rehabilitation in ZW (District B) also face urgent educational needs, which require non-District B families to go back to their local special education school. As a result, some families must choose between education and rehabilitation. The latest rehabilitation method for the integration of rehabilitation and education excludes children whose place of residence is different from their location of hukou, thus, creating an exclusion error.

*He cannot do rehabilitation next year. He has to go to school. The special education school in our hometown had been calling us since last year. He will be old to go to school (case 10)*.

#### Waste of Sources in Promotion Tournament System

People do not understand why rehabilitation services for disabled children aged 0–6 years differ among age groups in different provinces and cities. The available services are determined not only by the local economic development, but also are closely related to promotion tournaments, the third and distinctly informal rule in the regulatory context. In order to encourage each unit within the five management layers in China to work effectively, a competition mechanism has been formed in which the competition depends on a performance measurement. Distinguishing it from many Western countries, China's performance measurement of the unit is actually an evaluation of the principal person in charge of the unit (48), meaning that leaders of local organizations with good performance in the RASDC greatly enhance their chances of political advancement. The CDPF's leaders at all levels are motivated to gain a competitive advantage by increasing the number of beneficiaries of the RASDC.

The Disabled People's Federation in City J has broadened the policy criteria for outstanding performance in the assessment process. First, the restrictions on the financial conditions of the applicant's family have been canceled, and full coverage is available for all children with disabilities. Second, the age for eligibility of 0–6 years has been broadened to 0–17 years. However, as the director of the administrative office in ZW has demonstrated, the best time to rehabilitate children with disabilities is during the age range of 0–6 years, with children above the age of 7 not experiencing as great an effect from rehabilitation training as children aged 6 years and under do. Therefore, extending the age for eligibility leads to an inclusion error, and to some extent results in a waste of resources. Nevertheless, this conclusion does not mean that rehabilitation services for disabled children aged 7–17 should be canceled. The authors' viewpoint favors providing the older children with services that are more responsive to their specific needs, and thus, realizing an optimal allocation of resources.

*For children with disabilities aged 10-17, to be honest, their recovery in rehabilitation will be much worse. Actually, many have given up the rehabilitation. But some parents think that since it is a free service, they should enjoy it, at least the children can have a place to play, with the teacher caring [for] them. But they're crowding out other younger children*'s *resource (case 15)*.

### Normative Context: Strong Norms and Weak Social Responsibility in Rehabilitation Agencies

The normative dimension comprises the social norms and social responsibilities necessary for implementation of the RASDC. Social norms dictate how the policy implementation should be completed and determine the legal methods required to achieve the policy objectives, whereas, social responsibility refers to the preferred values in policy implementation and the attitude toward the obligations that are involved. The normative dimension illustrates the values of the rehabilitation agencies as the basic policy provider for children with disabilities.

#### Double Errors in Strong Norms

The Communist Party of China's Central Committee and the State Council pay great attention to the RASDC and provide a strong and widely shared norm for rehabilitation agencies, which are the firstline service providers. In present-day China, private non-enterprise units, or non-governmental organizations (NGOs), are often not spontaneous grassroots organizations but instead are heavily influenced by China's policy support and financial support, and therefore, tend to be bureaucratic. That system results in repeated, organized, and institutional norms that emphasize policy outcomes, and such an emphasis creates strong external pressure on the agencies – which are highly dependent on external resources and have the primary task of responding to the requirements of those external organizations. That systemic pressure is not always realistic or effective – on the contrary, in many cases, it only completes the purpose of “ritual” without really meeting the users' needs. In particular, different rehabilitation agencies face different pressures, and the types of deviations that result are also diverse. Our findings revealed that the children's hospital had the largest demands and could only complete its vested tasks through formal or informal methods, such as, modifying standards, screening classifications, and limiting queuing, thus, producing an exclusion error. However, the demands of backward areas or newly established rehabilitation agencies are often small, and they have to fulfill their official tasks by reducing the criteria for eligibility and attracting additional children, thus, generating an error of inclusion. One manager we interviewed described the phenomenon and her ideas about the RASDC.

*Free rehabilitation assistance in children's hospitals is limited on quotas. The quota is now 6 months away. The children will be given only charged items after they are diagnosed at the children's hospital. Some parents know that they can go to other agencies, but those who don't know have to go home (case 15)*.

#### Selective Neglect in Situations of Weak Social Responsibility

Although, rigid norms have been the guidelines for policy implementation, the criteria for comparing and evaluating the behavior of the RASDC's implementors have been insufficient, and that deficiency has caused rehabilitation agencies to lack social responsibility. Rehabilitation services for children with disabilities currently are provided by a form of “government procurement services.” The entities that undertake the government purchasing services are mostly NGOs, which by definition are social organizations organized by enterprises, social groups, and other social forces, as well as individual citizens, using non-state-owned assets to engage in non-profit social service activities. As NGOs, those non-profit organizations still have a “for-profit” need to maintain the operation of the organization. In ZW, “90% of the revenue comes from the support of the RASDC program.” Managers complained more than once in our interviews about funding challenges, such as, insufficient government money and unclear government requirements.

One lesson from 2020 has made the head of ZW very cautious this year. That year, a new model, “institution + family + community” rehabilitation, was introduced for children who are more seriously disabled and unable to leave their home. In order to obtain additional public funds, ZW cooperated with a special education school to render rehabilitation services in children's homes. However, the money failed to be delivered at the end of the year, because 3-month centralized institution-based rehabilitation had been lacking (the criteria were emphasized at the time of the assessment). Now, ZW has given up that group of children reluctantly. Clearly, the economic demands of the agency far exceeded the level of social responsibility of the rehabilitation services. The primary aim of the agency was to obtain, as far as possible, children with disabilities who were easily rehabilitated and could acquire the funds from the RASDC. For other children with higher degrees of disability, and inability to provide them with institution-based rehabilitation, and an attitude of neglect, have been adopted toward them. In practice, this behavior that the agencies engaged in to further their own interests was obviously inconsistent with the original purpose of the well-targeted RASDC and has led to numerous cases of exclusion errors.

In addition, no criteria have been developed for evaluating policy implementation from the perspective of the disabled children and their households. On the one hand, the local government's management strategies, from incentives to penalties, still seem focused on shaping the rehabilitation agencies into disciplined and efficient implementors of official discourse and have not offered much autonomy or flexibility for the families with disabled children. On the other hand, influenced by difficulties with social supervision, inadequate development of citizens' participation in politics, and a negative disability culture, few people have the opportunity and willingness to pay attention to the RASDC's implementation. The absence of supervision from service users and the public has further reduced the level of social responsibility in rehabilitation institutions, and that situation fosters an exclusion error. The direct evidence from the managers' narratives reveals that:

*Now it is mainly [up to] the Disabled Persons' Federation, or a third-party organization invited by the Disabled Persons' Federation, to supervise. They don't understand at all, fill out the form and they're done (case 14)*.

### Cognitive Context: Identity Culture in Families of Disabled Children

In traditional Chinese society, referring to someone as “canji” or “canfei” means the person is disabled and cannot do anything. These words have obvious derogatory and discriminatory implications and do not place the disabled and the abled on equal footing (49). This notion of inequality about persons with disabilities further influences attitudes on procreation and care, and in so doing leads to another implementation deviation of the RASDC.

#### The Exclusive Effect of Stigmatization of Children With Disabilities

In ancient China, persons with disabilities did not possess the qualifications to enjoy complete rights. For example, a person with a disability was not allowed to enter the ancestral hall and not allowed to be an official — rules that treated persons with disabilities differently from other people (50). Furthermore, the Chinese words describing disability, such as, “xiazi” (visual disability), “longzi” (hearing disability), “yaba” (speech disability), “quezi” (physical disability), “shazi” (intellectual disability), and “fengzi” (mental disability), are cited in everyday language as acceptable material for teasing, blaming, and reviling, even to the present day. These words are labels that carry serious stigma and create psychological obstacles in the parents. Welfare recipients are often culturally stigmatized; thus, they are not willing to admit that their children are disabled and they are reluctant to receive rehabilitation services that identify the children as being disabled. These concepts lead to subjective exclusion by service recipients.

*In [our] village, only my child receives rehabilitation service. Others do not and their parents reject rehabilitation, because they don't admit that their child is disabled. They take it to be natural when the child cannot speak at three (case 5)*.

*The child of my wife's classmate is also autis[tic]. My wife told her to take her child for rehabilitation in City J, saying that our child is getting better in the ZW. My wife told her once or twice, and later she [wa]s obviously out of touch with us, without coming to our home or talking to us on the way. She feels that we look down on her by always saying that her child is disabled (case 6)*.

One condition by which children with disabilities can obtain free rehabilitation services is to have a disability license, which is an official document for people with disabilities composed of their ID number and two other numbers that refer to their grade of disability and their type of disability. In that negative environment, some parents reject the disability license, which is obtainable after a strict medical diagnosis. Even when they have the desire to get rehabilitation assistance for their children, they can be persuaded to quit because of this paper identification, thus, causing an exclusion error.

*His father is not willing to have the disability license, saying that the child will complain when he grows up. He feels that the disability card is a child's life shame (case 7)*.

In other cases, family members accept the fact that their children are disabled, but correspondingly, that kind of disability culture fosters a sense of inferiority and shame in those families. A family's interactions with others without disabilities play a significant role in constructing the family members' disabled identities and facilitating their negative perspective toward their lives. Female parents, especially, express fear or anger at the eyes of strangers in their workplace. They always reject parties with friends or relatives, feeling that they are “inferior” and “worse than others.” Under this self-cognition, their goal of rehabilitation for their children is to have normal children or children who appear to be normal. If the family members see no hope of recovery for the disabled child, they will refuse to receive rehabilitation.

*There is no hope to recovery. It is too tir[ing]. We want to send her to a care center. Once she is there, we can leave it alone and just go to see her once a month (case 9)*.

#### Choice of Care in the Traditional Concept of Procreation

The concept of procreation, under the nation's family planning policy, is closely linked to the idea that persons with disabilities are incomplete and inferior. A family planning policy has been carried out in China from 1982 to the present. From 1982 to 2015, a one-couple-one-child policy, or one child in one couple, was strictly enforced. In some special cases, however, two children were allowed, and one such special case was when the first child of the couple was disabled. Since 2016, a one-couple-two-children policy has been adopted, and in some special cases three children are allowed, including the case in which “the couple gives birth to two children, with one of them identified as being handicapped who cannot grow into a normal labor force according to law” (51). With the exception of some households that are afraid of having another child with a disability, most couples do choose to have another child and hope that he/she is normal. Otherwise, the family is not considered perfect.

In Chinese society, the family plays the essential role in providing welfare for disabled children in their living situation and their spiritual life. Throughout the nation's long historical development, the Chinese family system has always preserved great internal stability and formed a solid family care model (52). Research findings have pointed out that the fixed cycle, beginning with “raising children” and ending with “caring for the old,” is maintained and reproduced intergenerationally. Of the two parents, the mother is usually the caregiver during the stage of raising the children. In our study, in cases 5, 6, and 10, other family members undertook caregiving duties only because the mothers were disabled. Furthermore, in general, after the birth of the family's second or third child, the mother, as the major caregiver, cannot maintain a balance in taking care of two or even more children, one of whom is disabled. Then, she must choose between two options.

The first choice is to voluntarily abandon the disabled child and to give adequate care to the healthy one, thus, resulting in an exclusion error.

*The eldest brother is 23 now and is going to get married soon. I cannot stay here all the time. So next year, we plan to give up rehabilitation. We'll see whether we can send him to a special education school so that I'll have time to manage my family matters (case 11)*.

The second option is to depend on the system of urgently needed care agencies to provide care for the child with a disability. When care agencies and professional care workers are scarce and expensive, such families are forced to transfer the care pressure to rehabilitation agencies, wherein children can have classes every day. In that way, the care burden of the family can be alleviated, thus, resulting in an inclusion error.

*This child is 15 now. To be honest, he cannot make any progress here. He comes just to play. His mother and father are both working without any time to take care of him. I send him here every day. There's a teacher in class, and we don't have to take care of him every minute (case 4)*.

## Discussion and Conclusions

This research, based on a case study of ZW, in Mainland China, started with a three-dimensional theoretical framework for policy context and conducted a systematic analytic investigation into supply-demand deviations in the implementation of the RASDC, as well as the reasons behind those deviations. The study's major finding revealed that the failure of the rehabilitation policy was not due to just the policy content itself but was also due to the context of policy implementation, with special aspects of the Chinese environment affecting the final result of implementing the RASDC, which have a negative impact on their health. The empirical data we collected presented exclusion errors, in which those who should enjoy the services did not receive them, and also inclusion errors of wasted resources. To be concrete, in the regulatory context, the local government's implementation of the RASDC policy was affected by formal and informal rules, such as, the targeted management responsibility system, the administrative contracting system, and promotion tournaments, all of which caused barriers such as, the exclusion of disability types, exclusion due to inconsistent service availability in the region of the family's residence, and inclusion errors of extra services being given to those who did not need them. In the normative context, the behavior and activity of rehabilitation agencies were limited by strong norms and weak social responsibility. Priority was given to accomplishing the government's tasks formally, not virtually, by showing preference to those with mild disabilities and neglecting those with severe disabilities. Finally, in the cognitive context, children with disabilities were considered as a special group, and were conceived of as being different from healthy people. Households with disabled children felt ashamed and inferior. Those kinds of attitudes prevented the families from actively acquiring the services, or forced them to relinquish the services, or pressured them to transfer the disabled child's care in the dynamic of continuing to have other children. These findings are summarized in Table 5.

**Table 5:** Contexts and types of deviations.

**Contexts**	**Characteristics of contexts**	**Types of deviations**
Regulatory context	Target management responsibility system	Exclusion error
	Administrative subcontract system	Exclusion error
	Promotion tournament system	Inclusion error
Normative context	Strong norms	Exclusion error, inclusion error
	Weak social responsibility	Exclusion error, inclusion error
Cognitive context	Stigmatization	Exclusion error
	Traditional concepts of procreation & care	Exclusion error, inclusion error

This research can provide inspiration and suggestions for improving the precision of implementing the RASDC policy's services for children with disabilities. The World Health Organization (WHO) is trying to design international standards for rehabilitation services that can be used everywhere, one of which is *the WHO Global Disability Action Plan 2014-2021: better Health for All People with Disability*. The action plan puts forward 7 actions to achieve the goal of “strengthen and extend rehabilitation, habilitation, assistive technology, assistance and support services, and community-based rehabilitation”: providing leadership and management, providing adequate financial resources, developing and maintaining a sustainable workforce, establishing a health care system mechanism for effective coordination, making available appropriate assistive technologies, promoting access to a range of assistance and support services, supporting and building the capacity of persons with disabilities, and their family members and/or informal caregivers (53). According to the findings of this article, combined with the international reform plan, rehabilitation policy in Mainland China needs to be optimized and reduce both exclusion errors and inclusion errors, so as to realize the vision of accurate rehabilitation for all person with disabilities. This action plan specifically includes:

Proposed action 1: Revise policies, standards, and implementation managements. First, the government should strengthen policies, strategies and plans on habilitation, rehabilitation, assistive technology, support and assistance services, community-based rehabilitation, and related strategies based on the actual number and needs of disabled children. Secondly, the government should break geographical boundaries and provide adequate financial resources to increase coverage and access to rehabilitation services for non-local children with disabilities. Finally, the evaluation indicators of the Disabled Persons' Federations at all levels should be reformed by comprehensively evaluating the actual service demand and service quality, rather than the policy texts and supply quantity.

Proposed action 2: Enhance the social responsibility of rehabilitation agencies. To begin with, a rehabilitation assistance network should be built to exchange information between different rehabilitation agencies and, when appropriate, to provide referral services in order to balance the different pressure of rehabilitation agencies. Moreover, third-party supervision and bottom-up public supervision should be strengthened to improve rehabilitation agencies' social responsibility and to achieve a rational allocation of rehabilitation resources. In addition, it is important to train health personnel for early identification, assessment, and referral of children with disabilities, especially in undeveloped areas.

Proposed action 3: Foster an environment of equality, social acceptance, and integration for children with disabilities. We need to internally and culturally change the minds, feelings, and acquired dispositions of the public and families with disabilities, or, in Bourdieu's word, their habitus. During the transformational process, it will be important to build social consensus by publicizing rehabilitation through networks and the media, by popularizing the New Concept toward Persons with Disabilities, and by enhancing people's social sense of responsibility. We also must increase communication with families who have children with disabilities and convince them that those children are not just a drag on the family – that instead, their children can return to their families and society through proper rehabilitation treatments. Psychological support provided for parents with disabled children will improve the quality of care they give to their children and consequently increase the quality of life of children and family (54).

This research had three main limitations. First, it was difficult to obtain much information from the past research, because this was the first qualitative research discussing implementation deviation of the RASDC, and the reasons for it, from the perspective of policy context. Second, the RASDC was a new policy, and there were fluctuations in the task indexes and evaluation methods that might have affected the credibility of the data. Last, this research was based on a single case study, but China's population structure, its urban and rural structures, and its bureaucratic structure are quite complex, meaning that the RASDC differs in different provinces and cities. As a result, the applicability of this study's conclusions to other regions and situations needs further discussion and reflection. In the future, additional qualitative and quantitative research should be conducted to extend the credibility of our existing analysis.

## Data Availability Statement

The original contributions presented in the study are included in the article/supplementary material, further inquiries can be directed to the corresponding author/s.

## Ethics Statement

The studies involving human participants were reviewed and approved by Academic Committee of Jilin University. The patients/participants provided their written informed consent to participate in this study.

## Author Contributions

CQ: conceptualization, methodology, investigation, writing the original manuscript, revising the manuscript, and administration. YW: conceptualization, investigation, writing the original manuscript, and revising the manuscript. All authors: contributed to the article and approved the submitted version.

## Conflict of Interest

The authors declare that the research was conducted in the absence of any commercial or financial relationships that could be construed as a potential conflict of interest.

## Footnotes

^1^It should be noted that ages 0–6 years are not the absolute age limit. In fact, China's 31 provinces (districts, municipalities) and Xinjiang Production and Construction Corps have introduced local RASDC according to local conditions, and among those provinces, 17 provinces (districts, municipalities) have widened the age limit for eligible applicants to varying degrees (data from 2019), such as, ages 0–15 years in Beijing and 0–17 years in Shanghai. City J, the place for investigation in this paper also extended the eligible ages to 0–17 years.
